# Surgical experience with caseous calcification of the mitral annulus: a case report

**DOI:** 10.1093/jscr/rjag604

**Published:** 2026-07-16

**Authors:** Kazuki Mori, Masato Morita, Eisuke Kawakubo, Masazumi Kume, Yuichi Nakazono, Takashi Shuto

**Affiliations:** Department of Cardiovascular Surgery, NHO Beppu Medical Center, 1473 Uchikado, Beppu, Oita 874-0011, Japan; Department of Cardiovascular Surgery, NHO Beppu Medical Center, 1473 Uchikado, Beppu, Oita 874-0011, Japan; Department of Cardiovascular Surgery, NHO Beppu Medical Center, 1473 Uchikado, Beppu, Oita 874-0011, Japan; Department of Cardiovascular Surgery, NHO Beppu Medical Center, 1473 Uchikado, Beppu, Oita 874-0011, Japan; Department of Pathology, NHO Beppu Medical Center, 1473 Uchikado, Beppu, Oita 874-0011, Japan; Department of Cardiovascular Surgery, Oita University, 1-1 Idaigaoka, Hasama-machi, Yufu, Oita 879-5593, Japan

**Keywords:** caseous calcification, mitral annular calcification, hemodialysis

## Abstract

Caseous calcification of the mitral annulus (CCMA) is a rare variant of mitral annular calcification associated with an increased risk of embolic stroke and diagnostic difficulty. We report a surgical case of CCMA that showed rapid enlargement after the initiation of hemodialysis. A 71-year-old woman undergoing hemodialysis for 1 year presented with a rapidly enlarging mass at the P3 segment of the posterior mitral annulus. Echocardiography and computed tomography revealed a calcified mass with a hypoechoic/low-attenuation core. Intraoperatively, the lesion contained creamy caseous material, and mitral valve replacement with annular reconstruction was required after debridement. Histopathology confirmed CCMA without malignancy or infection. CCMA may progress rapidly in patients on hemodialysis, likely due to disordered calcium–phosphorus metabolism and chronic inflammation. Early surgical intervention with complete isolation of caseous material may help prevent systemic embolism.

## Introduction

Caseous calcification of the mitral annulus (CCMA), a tumor-like lesion of the mitral annulus, is characterized by a calcified shell filled with internal caseous material. It is a rare variant of mitral annular calcification (MAC) that reportedly occurs in ~0.6% of patients with MAC [1]. Patients with CCMA have a high incidence of embolic stroke during their clinical course [2]. Herein, we present a surgical case of CCMA that demonstrated significant enlargement over a 1-year period.

## Case report

**Figure 1:**
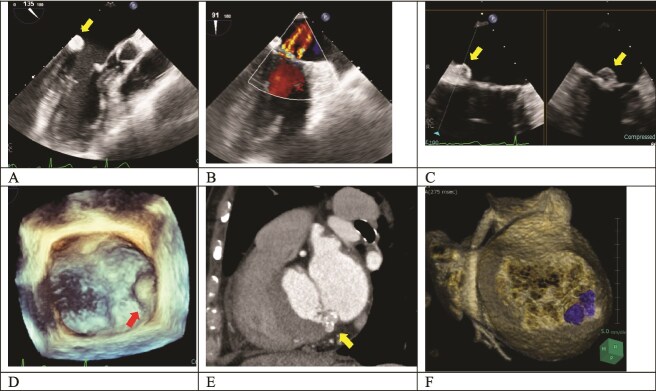
Preoperative images. (A) Echocardiography showing a hyperechoic mass (arrow) at the posterior leaflet of the mitral valve. (B) Color Doppler echocardiography demonstrating mild mitral regurgitation. (C) A mass lesion (arrows) with an internal hypoechoic core is observed attached to the posterior mitral annulus, accompanied by acoustic shadowing. (D) A protruding lesion (arrow) was observed within the left atrium at the P3 segment of the mitral valve on the reconstructed image. (E) CT showing a calcified mass (arrow) with an internal low-attenuation area. (F) Three-dimensional reconstruction image revealing a mass lesion occupying the posterior mitral annulus (P3 segment).

A 71-year-old woman undergoing hemodialysis for 1 year presented with a mass lesion on the mitral valve during routine transthoracic echocardiography. Echocardiography performed 1 year before hemodialysis initiation showed no evidence of the lesion. Laboratory examinations revealed no elevation of inflammatory markers suggestive of infectious endocarditis. Meanwhile, echocardiography showed a 10 × 12 mm hyperechoic mass located at the P3 segment of the posterior mitral leaflet annulus, with associated mild mitral regurgitation (Fig. 1A–D). Based on computed tomography (CT), the mass was characterized by peripheral calcification (Fig. 1E and F). Moreover, the mass exhibited internal low attenuation without any significant enhancement. Brain magnetic resonance imaging (MRI) revealed several old lacunar infarctions in the cerebral white matter.

**Figure 2:**
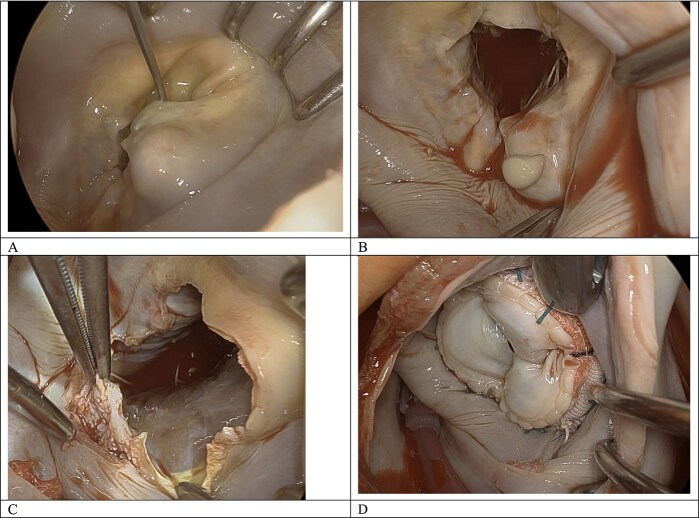
Intraoperative findings. (A) An elastic, hard, and smooth-surfaced protruding mass is observed at the P3 segment of the mitral annulus. (B) After lesion incision, creamy caseous material was discharged from the interior of the lesion. (C) Mitral annulus showing a defect after thorough debridement of the caseous contents. (D) Mitral valve replacement combined with annular reconstruction using an autologous pericardial patch.

Cardiopulmonary bypass was established via median sternotomy using ascending aortic and bicaval cannulation. Left atrial appendage exclusion was performed using AtriClip (AtriCure, Inc., Mason, OH, USA). Cardioplegic arrest was achieved by antegrade cardioplegia and maintained with subsequent retrograde cardioplegia. The mitral valve was exposed through a superior transseptal approach. An elastic, smooth-surfaced mass was identified at the P3 segment of the posterior mitral annulus (Fig. 2A). With no valvular destruction or inflammation, mass incision evacuated creamy material (Fig. 2B). After debridement of peripheral calcification using an ultrasonic surgical aspirator and subsequent irrigation, an annular deficiency was revealed (Fig. 2C). Although the mitral valve leaflets showed no destruction, they were detached from the annular margin. Mitral valve replacement was performed to prioritize long-term stability since simple patch closure carried a risk of recurrence and the mitral leaflets exhibited mild rheumatic-like thickening. The mitral valve’s anterior and posterior leaflets were completely resected, and the annular defect was patched with autologous pericardium. Mitral valve replacement was performed using a 29-mm Epic Plus (Abbott, Chicago, IL, USA) (Fig. 2D). After confirming the absence of perivalvular leakage, the patient was successfully weaned from cardiopulmonary bypass. Intraoperative findings are shown in the Supplementary Video S1.

**Figure 3:**
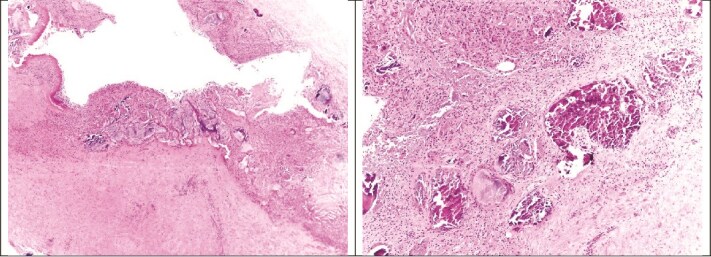
Histopathological findings (hematoxylin and eosin staining). The specimen showed fine calcified deposits and a foreign body response characterized by neutrophilic and lymphocytic infiltration alongside macrophage clustering. No evidence of bacterial pathogens or malignancy was observed.

Bacterial cultures of the lesion contents were negative. Pathological examination revealed fine calcified deposits and a foreign body response with neutrophilic and lymphocytic infiltration and macrophage clustering. Notably, we did not observe bacterial pathogens nor malignant features (Fig. 3). A final diagnosis of CCMA was made through a combination of surgical and pathological evidence.

The patient resumed hemodialysis on postoperative day 2 and received intensive care unit treatment for 4 days after the surgery. No complications occurred during the hospital stay. Postoperative echocardiography showed no evidence of perivalvular leakage or valvular impairment.

## Discussion

CCMA, a rare variant of MAC, typically presents as a tumor-like lesion characterized by a central core of putty-like material, which is an admixture of fatty acids, cholesterol, and calcium. In a large echocardiographic cohort, Deluca et al. reported that the prevalence of CCMA among patients with MAC was 0.6% [1]. The prevalence of CCMA is particularly high in patients with end-stage renal disease. This is because hemodialysis is a known factor that accelerates the condition, with numerous reports indicating progression following dialysis initiation [3–5]. Impaired calcium–phosphorus metabolism and persistent chronic inflammation are thought to be implicated in the accelerated calcification and subsequent liquefaction of the mitral annulus.

The potential for rapid progression in CCMA underscores the importance of differentiating it from infectious endocarditis, periannular abscesses, cardiac tumors, and intracardiac thrombi [3, 6]. Our patient developed a rapidly enlarging CCMA within one year of starting hemodialysis, showing typical caseous features: iso-to-hypoechoic on echocardiography and low-density with peripheral calcification on CT [3, 7]. The typical MRI appearance of CCMA includes a central high-intensity signal with a hypointense edge on T1-weighted images. Meanwhile, it is characterized by a central signal void surrounded by a hyperintense peripheral ring on T2-weighted images, often showing late gadolinium enhancement [6]. While absent inflammatory signs help distinguish CCMA from infective endocarditis, caution is necessary as CCMA can be complicated by infective endocarditis [8–10].

Despite unestablished surgical indications, CCMA often requires surgery to prevent devastating embolic stroke [2, 11]. Some reports have documented cases where CCMA resulted in conduction disturbances, leading to surgical intervention precipitated by atrioventricular block [12]. Therefore, early surgical intervention should be considered even in the absence of significant valvular dysfunction. CCMA’s mechanisms of embolism are thought to be attributed to the leakage of caseous contents, debris from necrotic tissues, and deposition of calcium and cholesterol components [8, 13]. Studies have also reported cases where CCMA complicated by infection led to devastating systemic embolism [10]. In the present case, early surgical intervention was opted because the rapid expansion observed over 1 year suggested an increased risk of spontaneous rupture. The intraoperative findings validated our concerns: the lesion was found to be under significant internal pressure, with the creamy material being easily expressed upon incision.

Definitive treatment necessitates thorough debridement of the caseous contents and associated calcification to eliminate the source of the chronic inflammatory response. In cases where the native mitral leaflets remain intact after debridement, direct suturing or autologous pericardial patch repair can be considered to maintain valvular function [4, 11]. When preserving the native valve, any residual inflammatory material may pose a risk for CCMA recurrence. Therefore, meticulous attention must be paid to ensure complete debridement [9]. In this case, thorough debridement created a significant annular defect. Therefore, mitral valve replacement combined with autologous pericardial patch reconstruction was performed to ensure structural integrity and durability, as the leaflets showed stenotic degeneration [7, 14]. Although a bioprosthesis was selected considering the patient’s age, it carries a significant risk of early structural valve deterioration under impaired calcium–phosphorus metabolism, necessitating rigorous long-term follow-up.

## Supplementary Material

Supplementary_video_rjag604
